# P-110. Promoting Optimal Treatment for Community-Acquired Pneumonia in the Emergency Room: a Before-After Study of a Novel Care Pathway

**DOI:** 10.1093/ofid/ofae631.317

**Published:** 2025-01-29

**Authors:** Jeffrey Pernica, April J Kam, Mohamed Eltorki, Joycelyne Ewusie, Sarah Khan, David M Goldfarb, Fiona Smaill, Shakeap Elliott, Jacqueline Wong, Melani Sung, Marek Smieja, Dominik Mertz, Lehana Thabane, Mark Loeb

**Affiliations:** Department of Pediatrics, McMaster University, Hamilton, Ontario, Canada; McMaster University, Hamilton, Ontario, Canada; McMaster University, Hamilton, Ontario, Canada; St Joseph's Healthcare Hamilton, Hamilton, Ontario, Canada; McMaster University, Hamilton, Ontario, Canada, Hamilton, Ontario, Canada; University of British Columbia, Vancouver, British Columbia, Canada; McMaster University, Hamilton, Ontario, Canada; Hamilton Health Sciences, Hamilton, Ontario, Canada; McMaster University, Hamilton, Ontario, Canada; Hamilton Health Sciences, Hamilton, Ontario, Canada; McMaster University, Hamilton, Ontario, Canada; McMaster University, Hamilton, Ontario, Canada, Hamilton, Ontario, Canada; McMaster University, Hamilton, Ontario, Canada; McMaster University, Hamilton, ON, Hamilton, Ontario, Canada

## Abstract

**Background:**

Community-acquired pneumonia (CAP) in young children is mainly caused by viral pathogens; for this reason, Infectious Disease Society of America guidelines explicitly state that antibiotic treatment of non-severe CAP in this population is not obligatory. However, the vast majority of non-severe pediatric CAP episodes diagnosed in North America prompt antibiotic prescription anyway.

**Figure:**
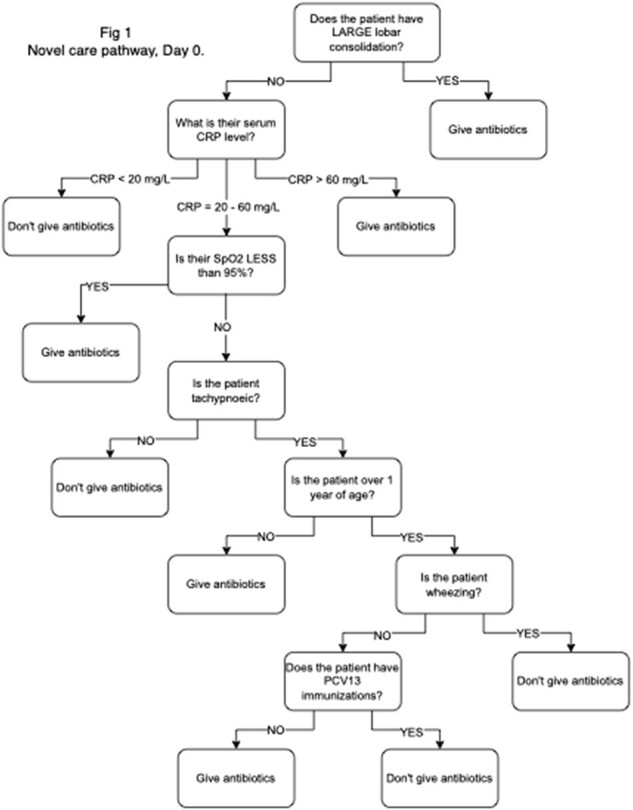

**Methods:**

A before-after prospective study was done in a Canadian emergency department (ED) enrolling children aged 6+ months without comorbidities diagnosed with CAP and managed as outpatients. In the before (control) phase, participants were managed as per routine care. In the after (intervention) phase, participants were managed using a novel care pathway. This care pathway used imaging results, a point-of-care C-reactive protein (POC CRP) assay, and other already-available data (eg. O2 saturation, respiratory rate, age) to categorize participants as ‘appreciable risk’ or ‘low risk.’ Those at ‘appreciable risk’ were given a prescription for antibiotics, whereas those at ‘low risk’ were simply discharged home. The next day, caregivers of ‘low-risk’ participants were informed of results of nasopharyngeal viral/atypical pathogen testing. Follow-up was done at day 2-5, 14-21, and 30.

**Figure:**
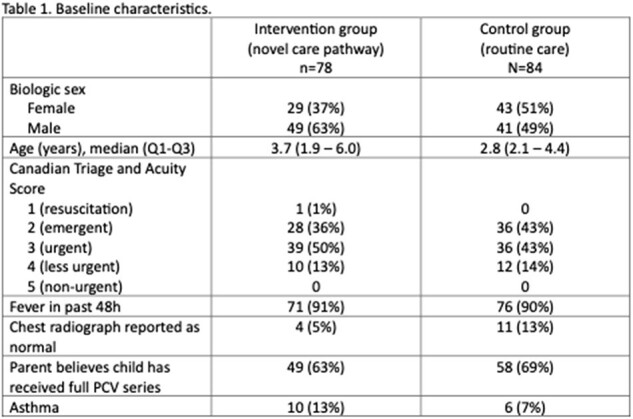

**Results:**

There were 84 participants recruited in the control phase (Mar-Nov 2022) and 78 in the intervention phase (Nov 2022-Mar 2024). Control phase participants had a median age of 2.8 y (IQR 2.1 – 4.4y) and 13% had normal chest imaging, whereas intervention phase participants had a median age of 3.7 y (IQR 1.9 – 6 y) and 5.1% had normal chest imaging. All 84 of the participants in the control phase were prescribed antibiotics at ED discharge, as compared to only 51% of those in the intervention phase (49% less, 95%CI 38-60% less, p< 0.001). There were no significant differences in clinical cure at day 2-5, re-presentation to the ED, later hospitalization, or caregiver satisfaction with the care plan.

**Conclusion:**

A simple novel care pathway incorporating POC CRP testing as well as other available data facilitated a very clinically important decrease in antibiotic prescribing without compromising clinical outcomes. This care pathway should be evaluated in the context of a multicentre randomized trial.

**Disclosures:**

**Jeffrey Pernica, MD, MSc, FRCPC, DTMH**, MedImmune: Grant/Research Support|Merck: Grant/Research Support **Dominik Mertz, MD, MSc**, KCI Inc. USA: Grant/Research Support

